# Enhanced Risky Choice in Male Rats Elicited by the Acute Pharmacological Stressor Yohimbine Involves Prefrontal Dopamine D1 Receptor Activation

**DOI:** 10.1093/ijnp/pyae006

**Published:** 2024-01-12

**Authors:** Alexandra Münster, Julia Huster, Susanne Sommer, Corinna Traxler, Angeline Votteler, Wolfgang Hauber

**Affiliations:** Systems Neurobiology Research Unit; Systems Neurobiology Research Unit; Department of Neurobiology, University of Stuttgart, Stuttgart, Germany; Systems Neurobiology Research Unit; Department of Neurobiology, University of Stuttgart, Stuttgart, Germany; Systems Neurobiology Research Unit

**Keywords:** Decision-making, risk, prelimbic cortex, dopamine, rat

## Abstract

**Background:**

Acute stress alters risk-based decision-making; however, the underlying neural and neurochemical substrates are underexplored. Given their well-documented stress-inducing effects in humans and laboratory animals, glucocorticoids such as cortisol and corticosterone and the α_2_-adrenoceptor antagonist yohimbine represent potent pharmacological tools to mimic some characteristics of acute stress.

**Methods:**

Here, we analyzed the effects of the pharmacological stressors corticosterone and yohimbine given systemically on risk-based decision-making in male rats. Moreover, we investigated whether pharmacological stressor effects on risk-based decision-making involve dopamine D1 receptor stimulation in the dorsal prelimbic cortex (PL). We used a risk discounting task that requires choosing between a certain/small reward lever that always delivered 1 pellet and a risky/large reward lever that delivered 4 pellets with a decreasing probability across subsequent trials.

**Results:**

Systemic administration of yohimbine increased the preference for the risky/large reward lever. By contrast, systemic single administration of corticosterone did not significantly promote risky choice. Moreover, co-administration of corticosterone did not enhance the effects of yohimbine on risky choice. The data further show that the increased preference for the risky/large reward lever under systemic yohimbine was lowered by a concurrent pharmacological blockade of dopamine D1 receptors in the PL.

**Conclusions:**

Our rodent data provide causal evidence that stimulation of PL D1 receptors may represent a neurochemical mechanism by which the acute pharmacological stressor yohimbine, and possibly nonpharmacological stressors as well, promote risky choice.

**Significance Statement**

Acute stress can impair decision-making under risk; however, little is known about the neural and neurochemical mechanisms involved. Here, we found that systemic administration of yohimbine, a pharmacological tool to mimic acute stress, increased risky choice in rats. Importantly, increased risky choice under systemic yohimbine was reduced by a concurrent blockade of prelimbic dopamine D1 receptors. Thus, excessive stimulation of prelimbic dopamine D1 receptors may be one neurochemical mechanism through which the pharmacological stressor yohimbine promotes risky choice. Possibly, excessive stimulation of prelimbic D1 receptors is also a mechanism through which nonpharmacological, naturalistic stressors alter decision-making under risk. These findings advance our understanding of the neural and neurochemical underpinnings of decision-making and provide insight into how risk-taking could be become increased under acute stress exposure.

## INTRODUCTION

Risk-based decision-making refers to the ability to choose between competing courses of action based on their benefits, such as the magnitude of the expected reward, and costs, such as the risk that a reward may not be forthcoming. In rodents, risk-based decision-making relies on a neural network including subregions of the prefrontal cortex, nucleus accumbens, and amygdala (Cardinal, 2006; Stopper and Floresco, 2011; Zeeb and Winstanley, 2011; St Onge et al., 2012; Paine et al., 2015; Zeeb et al., 2015; Orsini et al., 2018). Consistent with this notion, risk-based decision-making in humans is supported by the prefrontal cortex (Starcke and Brand, 2016) and impaired in patients with prefrontal damage (Manes et al., 2002). Moreover, a considerable body of evidence implicates the mesocorticolimbic dopamine (DA) system in various forms of decision-making (Schweimer and Hauber, 2005, 2006; Verharen et al., 2018), including risk-based decision-making (St Onge et al., 2011; Stopper et al., 2013; Mai et al., 2015; Piantadosi et al., 2021).

Acute stress can markedly alter decision-making under risk and uncertainty (Starcke and Brand, 2016). For instance, under acute stress, rats made more risky choices in a rodent Iowa gambling task (Nobrega et al., 2016). However, the effects of acute stressors on risky choice are complex and influenced by several variables, such as stressor identity (e.g., restraint, predator exposure in rodents), task structure (e.g., risk/uncertainty profile, working memory load), or sex (see Braun and Hauber, 2013; Bryce et al., 2020).

Given that acute stress stimulates the mesocorticolimbic DA system (Arnsten et al., 2012), risk-based decision-making and acute stress may share a common neurochemical substrate. Furthermore, increased risk-taking under acute stress is associated with enhanced noradrenergic (NA) activity (Montes et al., 2015). In addition, systemic administration of the stress hormone corticosterone promotes risky choice (Koot et al., 2013; Montes et al., 2015). These rodent findings are consistent with the general notion that acute stressors induce an orchestrated physiological response involving multiple stress mediators, such as catecholamines and glucocorticoids (Schwabe et al., 2022). However, they leave open the question through which brain areas and local neurochemical mechanisms acute stress mediators modify risk-based decision-making.

It is well known that the α2-adrenoceptor antagonist yohimbine increases NA cell firing in the locus coeruleus by blocking auto-receptors (Aghajanian and VanderMaelen, 1982). Through this mechanism of action, yohimbine elicits stress- and anxiety-like responses in many species, including (sub-)human primates, dogs, and rodents, and is being used as a major pharmacological stressor in numerous preclinical neuropsychiatric studies (Bremner et al., 1996a, 1996b). In addition, studies in humans and rodents on the actions of cortisol and corticosterone suggest that acute stress can enhance risk-taking through glucocorticoid-dependent mechanisms (van den Bos et al., 2009; Koot et al., 2013). These drugs are therefore considered to represent simple but potent pharmacological tools to mimic aspects of acute stressor effects on cognitive function (Schwabe et al., 2010).

In the present study, we sought to determine in more detail the neural and neurochemical basis of acute pharmacological stressor effects on risky choice. In Experiment 1, we analyzed the effects of yohimbine and corticosterone on risk-based decision-making in rats after single or combined systemic administration. To this end, we used a risk discounting task in which rats had to choose between a certain/small reward lever that always delivered 1 pellet and a risky/large reward lever that delivered 4 pellets with a decreasing probability in subsequent trials (Cardinal and Howes, 2005; St Onge and Floresco, 2009; Mai and Hauber, 2012, 2015). In view of evidence that NA activity alone or in concert with glucocorticoids mediates stressor effects on cognitive function (van Stegeren et al., 2007; Schwabe et al., 2010), we hypothesized that yohimbine would enhance risky choice, an effect that could be further augmented by co-administration of corticosterone.

Experiment 2 capitalizes on observations that prefrontal D1 receptors play a key role in mediating acute stress (Arnsten, 2015) and that yohimbine induces a co-release of NA and DA in several brain regions, including the prefrontal cortex (Devoto et al., 2006; Butts et al., 2011; Lemon and Manahan-Vaughan, 2012; Kempadoo et al., 2016). Therefore, we hypothesized that potential pharmacological stressor effects on risky choice assessed in Experiment 1 might be mediated, at least in part, by stimulation of prefrontal D1 receptors. To this end, we tested the effects of a prefrontal D1 receptor blockade in rats with and without concurrent systemic administration of pharmacological stressors. Microinfusions of the D1 receptor antagonist SCH23390 were targeted to the dorsomedial subregion of the prefrontal cortex (PL). This subregion encompasses the pregenual anterior cingulate cortex and dorsal prelimbic areas, which have been implicated in higher cognitive function and decision-making (Berridge and Devilbiss, 2011; Diehl and Redish, 2023).

## MATERIALS AND METHODS

All animal experiments were performed in accordance with the German Law on Animal Protection and approved by the proper authorities.

### Experiment 1: Effects of Yohimbine and Corticosterone on Risky Choice

#### Subjects

Male Lister hooded rats (Charles River, Sulzfeld, Germany) (n = 20) weighing between 200 and 225 g on arrival were used. They were housed in transparent plastic cages (60 cm × 38 cm × 20 cm, Tecniplast, Milan, Italy) in groups of up to 4 animals. Rats were maintained on 12-hour-light/-dark cycle (lights on at 7:00 am) with ad libitum access to water. Upon arrival, standard laboratory chow (Altromin, Lage, Germany) was given ad libitum for at least 5 days. Thereafter, food was restricted to 15 g per animal per day. For environmental enrichment, a plastic tube (20 cm, Ø 12 cm) was attached to the top of each cage. Temperature (22°C ± 2°C) and humidity (50% ± 10%) were kept constant in the animal house.

#### Apparatus, Habituation, and Lever Press Training

The equipment of the operant boxes is described in detail in Mai and Hauber (2015).

On the first day, a 15-minute session was conducted with free access to 25 pellets in the food magazine. Throughout the session, the chamber was illuminated by the house and stimulus lights. On the next day, a 30-minute session was conducted using an interval schedule with variable pellet delivery (mean interval, 60 seconds; minimum interval, 10 seconds; maximum interval, 110 seconds), signaled by the illumination of the stimulus light. On the following days, animals were trained to lever press for 2 consecutive days in 2 daily 15-minute sessions using a fixed ratio-1 schedule for one lever and then for the other lever. Each lever was extended throughout the session with the house light always on.

Lever pressing training was followed by training on a simplified version of the full task. The task consisted of 24 trials for each lever, with the order of lever presentation chosen pseudo-randomly. Each trial began with house and stimulus lights on and the levers retracted. After a nose poking response into the food magazine within 10 seconds, the stimulus light was extinguished and one of the levers extended. If the rat responded to the lever within 10 seconds, the lever retracted and a single pellet was delivered. Then, the house light was extinguished; the inter-trial interval was set at 35 seconds. Failure to respond within 10 seconds of either to start the trial with a nose poke response or to lever press resulted in a 35-second time-out period, during which all lights were turned off and levers retracted. Animals were removed once all 48 trials were completed or after 60 minutes had elapsed. Rats were trained for 3 days with 1 session per day. After training on the simplified version, the full version of the risk discounting task was introduced.

#### Risk Discounting Task

The risk discounting task used here was based on protocols described by Cardinal and Howes (2005) and St Onge and Floresco (2009) and applied in previous own studies (Mai et al., 2015). For each animal, 1 of the 2 levers was designated as being the “small/certain lever” and the other as the “large/risky lever.” This assignment remained constant for each animal throughout the study and was counterbalanced across rats. Choosing the small/certain lever always resulted in the delivery of 1 pellet, whereas choosing the large/risky lever resulted in the probabilistic delivery of 4 or no pellets.

Each session of the risk discounting task consisted of 72 trials divided into 4 trial blocks. Each trial block consisted of 8 forced trials were followed by 10 free choice trials. Each trial began with the house and stimulus lights on and the levers retracted. The stimulus light was extinguished after a nose poking response to the food magazine within 10 seconds. In forced trials, only 1 of the levers was extended in order to expose the rat to the current response option and its associated value. Four trials were given for each lever presented in subsequent left-right pairs, the position of the initial lever presented in a pair was chosen pseudo-randomly. To study choice behavior of rats, in free-choice trails, both levers were extended at the same time. Response to a lever in forced or free choice trials within 10 seconds leads to retraction of all levers and the delivery of a reward matching the associated probability. Multiple pellets were delivered 0.5 second apart. Then, the house light was extinguished and the inter-trial-interval, set at 40 seconds, started. If a rat did not respond within 10 seconds to start the trial with a nose poking response or did not respond to the lever within 10 seconds, a 40-second timeout was initiated with all lights extinguished and levers retracted. These trials were considered as trial omissions and were repeated. In choice trials, lever presses for the small/certain and large/risky lever were measured.

The probability to obtain the large/risky reward systematically declined across 4 subsequent trial blocks of the risk discounting task (100%, 50%, 25%, 12.5%). Animals were trained on the task until, as a group, they showed within-session risk discounting, that is, a high preference for the large/risky lever (>90%) in the 100% large reward probability block and a low preference for the large/risky lever (< 50%) in the 12.5% large reward probability block. Thereafter, testing across experiments was started.

#### Testing for Effects of Systemic Yohimbine and Corticosterone: Behavioral Procedures

Yohimbine (Sigma-Aldrich, Munich, Germany) was dissolved in distilled water, and corticosterone (Sigma-Aldrich) was dissolved in 5% DMSO in isotonic saline. Drug and vehicle injection volumes were 2 mL/kg (i.p.), respectively. Doses of yohimbine (3 mg/kg) and corticosterone (1, 3 mg/kg) selected have been shown in earlier studies to be behaviorally active (Johnson et al., 2006; Koot et al., 2013). Post-administration, rats were returned to their home cages for 30 minutes until behavioral testing because approximately 30 minutes is required for yohimbine (Shepard et al., 2004) and corticosterone (Koot et al., 2013) to become behaviorally active after i.p. injection.

For capacity reasons, that is, a limited number of operant boxes, 2 distinct subgroups of rats were tested in separate experiments that were carried out one after the other. Two subgroups of animals (n = 10 each) were subsequently trained in the risk discounting task for 15 days, with each subgroup used to assess the effects of specific drugs/drug combinations on risk discounting. In both subgroups, each rat received drugs treatments as given below with 1 treatment on 1 test day per week. Baseline training sessions without drug administration were conducted 4 d/wk.

Drugs were administered using a within-subject cross-over design, that is, one-half of the animals received drug or vehicle on the test day in week 1; this assignment was reversed on the test days in the subsequent week.

In subgroup I, we first tested the effects of a single administration of yohimbine (3 mg/kg, i.p.) vs vehicle and, thereafter, of a single administration of corticosterone (1, 3 mg/kg, i.p.) vs vehicle. In subgroup II, we tested the effects of a combined administration of yohimbine (3 mg/kg, i.p.) plus corticosterone (3 mg/kg, i.p.) vs vehicle. The order of drug testing and assignment to subgroups is given in Table 1.

**Table 1.** Drug Design (Experiment 1)

Subgroup I (n = 10)								
Week 1		Week 2	Week 3		Week 4	Week 5		Week 6
Yoh 3 mg/kg (n = 5)	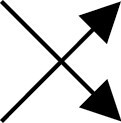	Yoh 3 mg/kg (n = 5)	Cort 1 mg/kg (n = 5)	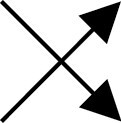	Cort 1 mg/kg (n = 5)	Cort 3 mg/kg (n = 5)	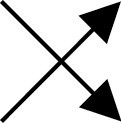	Cort 3 mg/kg (n = 5)
Veh 2 mL/kg (n = 5)		Veh 2 mL/kg (n = 5)	Veh 2 mL/kg (n = 5)		Veh 2 mL/kg (n = 5)	Veh 2 mL/kg (n = 5)		Veh 2 mL/kg (n = 5)
Subgroup II (n = 10)								
Week 1		Week 2						
Yoh 3 mg/kg + Cort 3 mg/kg (n = 5)	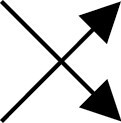	Yoh 3 mg/kg + Cort 3 mg/kg (n = 5)						
Veh 2 mL/kg (n = 5)		Veh 2 mL/kg (n = 5)						

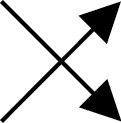
 = cross-over.

Abbreviations: Cort, corticosterone; Veh, vehicle; Yoh, yohimbine.

#### Data Analysis and Statistics

Percentage choices of the large/risky reward lever are given as means ± SEM. Within-subjects protocols were used for testing of subgroups 1 and 2; therefore, the data obtained from each subgroup were subjected to a repeated-measures ANOVA with 2 within-subjects factors (treatment, changing large reward probabilities = trial blocks). Because different rats were used in each subgroup, separate ANOVA were performed.

In the case of significant treatment effects on large/risky lever preferences, we also conducted an additional analysis to assess the impact of rewarded (“win”) and nonrewarded (“loss”) choices on subsequent choices. Specifically, we analyzed each choice as a function of the preceding choice in terms of reward or nonreward. These calculations were based on data from all free choice trials across the 4 blocks. The ratio of win-stay responses was calculated from the number of large/risky lever choices preceded by rewarded large/risky lever choices divided by the total number of large risky choices. Similarly, the ratio of lose-shift responses was computed from the number of small/safe lever choices preceded by nonrewarded large/risky lever choice divided by the total number of nonrewarded large/risky lever choices. Win-stay and lose-shift ratios were reported as means ± SEM. Respective ratios under drug vs vehicle were compared using the *t* test (2-tailed).

All statistical computations were carried out with STATISTICA (version 13.3, TIBCO, Palo Alto, CA, USA). The level of statistical significance (α-level) was set at *P* ≤ .05 (α-levels > .05 were designated as n.s., not significant).

### Experiment 2: Effects of Intra-PL D1 Receptor Blockade on Risky Choice

In Experiment 2, two other groups of animals were trained on the risk discounting task as described in Experiment 1 until the defined learning criterion was reached. The animals were then implanted with cannulae directed to the PL using standard stereotaxic procedures. Unless otherwise noted, the same behavioral procedures were used as in Experiment 1.

#### Surgery

Animals were anesthetized with ketamine (90 mg/kg, i.p.; Merial, Halbergmoos, Germany) and xylazine (4 mg/kg, i.m., Rompun, Bayer, Leverkusen, Germany). They were secured in a stereotaxic frame with atraumatic ear bars (Kopf Instruments, Tujunga, CA, USA) with the tooth bar set at −3.3 mm below the interaural line. The following coordinates were chosen with reference to the atlas of (Paxinos and Watson, 1997): AP +2.7 mm; ML ± 1.2 mm (angle 10°); DV −2.5 mm. Bilateral 11-mm-guide cannulae (0.8-mm outer diameter; Braun, Melsungen, Germany) were aimed at the PL. To prevent occlusion, stylets (0.45-mm outer diameter; Braun) were inserted into the guide cannulae. After surgery, rats received physiological saline (0.9%, 2 mL, s.c.) and the analgesic carprofen (Flunixin Meglumine 5%, 2.5 mg/kg, i.p., Pharma Partner, Hamburg, Germany). Animals were allowed to recover for at least 5 days. Thereafter, they were retrained on the risk discounting task for 4 days.

#### Intra-PL Microinfusion

Before microinfusion, animals were handled daily and, to familiarize them with the microinfusion procedure, infusion cannulae dummies (0.45-mm outer diameter; Braun, Melsungen, Germany) were lowered at the final infusion site on 2 days. Both infusion cannulae and dummies protruded 2 mm beyond the guide cannulae. In addition, 2 days before the first microinfusion, all animals received a bilateral infusion of physiological saline. On the microinfusion days, the stylets were removed and infusion cannulae connected to pump-driven microliter syringes (Med Associates, Fairfax, VT, USA) were introduced. Infusions (0.3 μL per hemisphere) were delivered bilaterally within 1 minute. After infusion, the infusion cannulae were left in place for an additional minute to allow for diffusion. Animals were then returned to their home cage for 15 minutes before being placed in the operant chamber.

#### Testing for Effects of Intra-PL SCH23390 Plus Systemic Pretreatment With Yohimbine: Behavioral Procedures

Two doses of intra-PL SCH2330 (1; 1.5 µg) vs intra-PL vehicle were examined in animals that had received systemic pretreatment with yohimbine (3 mg/kg, i.p.) in separate trials. Also, the effects of intra-PL SCH2330 (1; 1.5 µg) vs intra-PL vehicle were examined without systemic yohimbine pretreatment. Because Experiment 1 revealed that corticosterone alone had no effect on risky choice, we only tested the effects of systemic administration of yohimbine in combination with intra-PL microinfusion of SCH23390. The selected doses of SCH23390 were based on previous studies demonstrating behavioral efficacy after PL infusion (Calaminus and Hauber, 2008; Gonzalez et al., 2014). SCH23390 (Sigma Aldrich) was dissolved in physiological saline (0.9%). A within-subject design was used for drug and vehicle microinfusions.

For capacity reasons, that is, a limited number of operant boxes, 2 distinct subgroups of rats were tested in separate experiments carried out one after the other. Each subgroup was trained in the risk discounting task for 15 days, and then each subgroup was used to assess the effects of specific drugs/drug combinations on risk discounting. Microinfusions were conducted on 1 test day per week. Baseline training sessions without microinfusions were conducted 4 days per week. Drugs/drug combinations were administered using a within-subject cross-over design, that is, one-half of the animals received drug or vehicle on the test day in week 1; this assignment was reversed on the test days in the subsequent week.

In subgroup I, we tested the effects of intra-PL microinfusions of 2 doses of SCH23390 (1.0 and 1.5 µg) vs vehicle (0.3 µL) in animals that received systemic pretreatment with yohimbine (3 mg/kg, i.p.). In subgroup II, we subsequently tested the effects of intra-PL microinfusions of the same 2 doses of SCH23390 (1.0 and 1.5 µg) vs vehicle (0.3 µL) in animals that received systemic pretreatment with vehicle (2 ml/kg, i.p.) instead of yohimbine.

The order of drug testing in respective subgroups is given in Table 2. Because we used within-subjects protocols for testing of subgroup 1 and 2, the data obtained from both subgroups were subjected to a repeated-measures ANOVA with 2 within-subjects factors (treatment, changing large reward probabilities = trial blocks). Because different rats were used in each subgroup, separate ANOVA were performed.

**Table 2.** Drug Design (Experiment 2)

Subgroup I (n = 15)						
Route	Week 1		Week 2	Week 3		Week 4
Intra-PL	SCH23390 1.0 µg (n = 8)	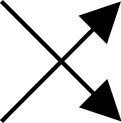	SCH 22390 1.0 µg (n = 7)	SCH23390 1.5 µg (n = 8)	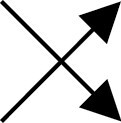	SCH22390 1.5 µg (n = 7)
Intra-PL	Veh 0.3 µL (n = 7)		Veh 0.3 µL (n = 8)	Veh 0.3 µL (n = 7)		Veh 0.3 µL (n = 8)
Intraperitoneal	Yoh 3 mg/kg (n = 15)		Yoh 3 mg/kg (n = 15)	Yoh 3 mg/kg (n = 15)		Yoh 3 mg/kg (n = 15)
Subgroup 2 (n = 13)						
Route	Week 1		Week 2	Week 3		Week 4
Intra-PL	SCH23390 1.0 µg (n = 7)	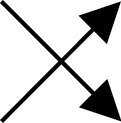	SCH 22390 1.0 µg (n = 6)	SCH23390 1.5 µg (n = 7)	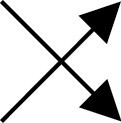	SCH22390 1.5 µg (n = 6)
Intra-PL	Veh 0.3 µL (n = 6)		Veh 0.3 µL (n = 7)	Veh 0.3 µL (n = 6)		Veh 0.3 µL (n = 7)
Intraperitoneal	Veh 2 mL/kg (n = 13)		Veh 2 mL/kg (n = 13)	Veh 2 mL/kg (n = 13)		Veh 2 mL/kg (n = 13)

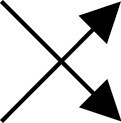
 = cross-over.

Abbreviations: Veh, vehicle; Yoh, yohimbine.

#### Histology

After completion of behavioral testing, all animals were euthanized with an overdose of isoflurane (Abbott, Wiesbaden, Germany) to check for correct cannula placement. Brains were removed, fixed in 4% formalin for 24 hours, and stored in 30% glucose. Brains were frozen and coronal brain sections (35–40 µm) were collected, mounted on coated slides, and stained with cresyl violet.

## RESULTS

### Experiment 1: Effects of Systemic Corticosterone and Yohimbine on Risky Choice

#### Effects of Yohimbine

**Figure 1.**
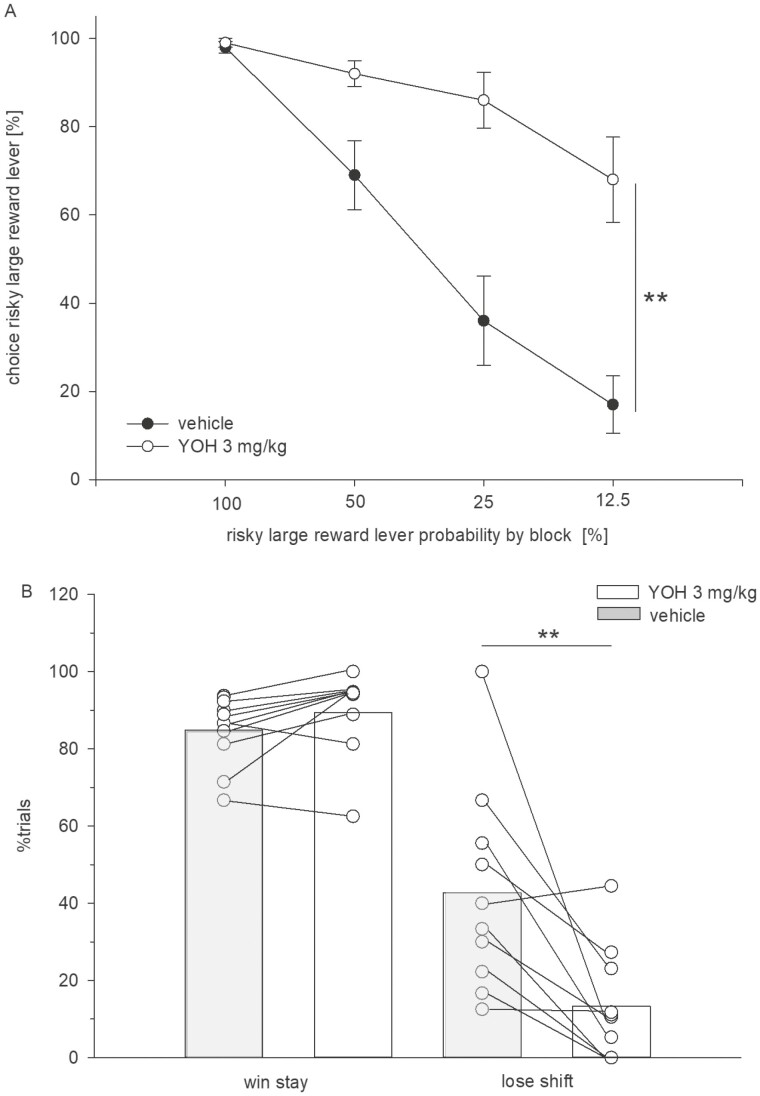
(A) Effects of yohimbine (3 mg/kg, i.p.) vs vehicle on probabilistic choice. Mean (± SEM) percentages of large/risky lever choices per session are shown across decreasing probabilities to obtain the large/risky reward within 1 session (n = 10). **P < .01 (significant main effect of treatment, ANOVA). (B) Mean proportion (±SEM) of large/risky lever choices after risky wins among all rewarded large/risky lever choices (“win-stay”) and mean proportion (± SEM) of small/certain lever choices after risky losses among all small/certain lever choices (“lose-shift”). **P < .01, *t* test.

Results demonstrated that, compared with vehicle, yohimbine (3 mg/kg, i.p.) increased the large/risky lever preference (Figure 1A). An ANOVA indicated a main effect of treatment (F_(1,9)_ = 43.37, *P* < .001) and trial block (F_(3,27)_ = 33.67, *P* < .001) and a significant treatment × block interaction (F_(3,27)_ = 14.27, *P* < .001). Win-stay ratios were not altered by treatment (*t*_9_ = 1.73, n.s.), whereas lose-shift ratios were markedly lower under yohimbine treatment (*t*_9_ = 3.43, *P* < .001) (Figure 1B).

#### Effects of Corticosterone

**Figure 2.**
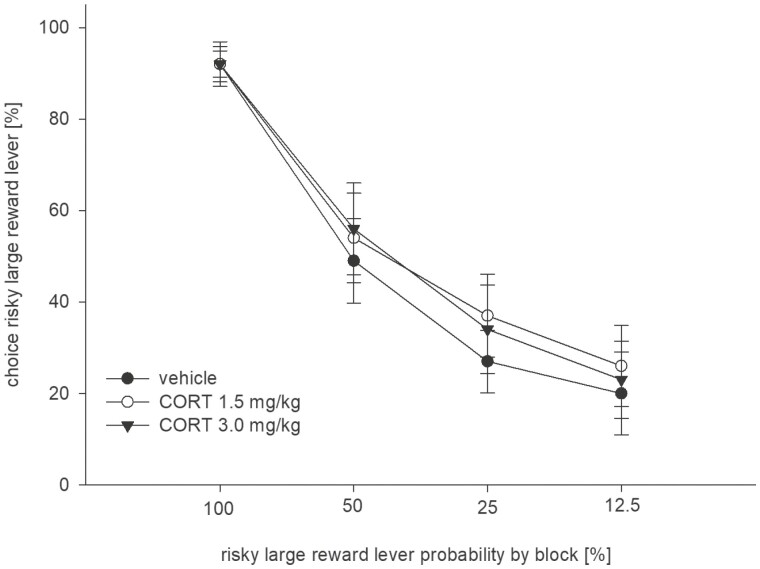
Effects of corticosterone (1.5, 3 mg/kg, i.p.) vs vehicle on probabilistic choice. Mean (±SEM) percentages of large/risky lever choices per session are shown across decreasing probabilities to obtain the large/risky reward within 1 session (n = 10).

Next, we examined the effects of corticosterone (1.5, 3 mg/kg, i.p.) against vehicle. Neither dose of corticosterone altered the preference for the large/risky lever (Figure 2). Consistent with this description, an ANOVA revealed no main effect of treatment (F_(2,18)_ = 1.51, n.s.), a significant effect of trial block (F_(3,27)_  = 53.30, *P* < .001), and no treatment × block interaction (F_(6,54)_  = 0.45, n.s.).

#### Effects of Co-administration

**Figure 3.**
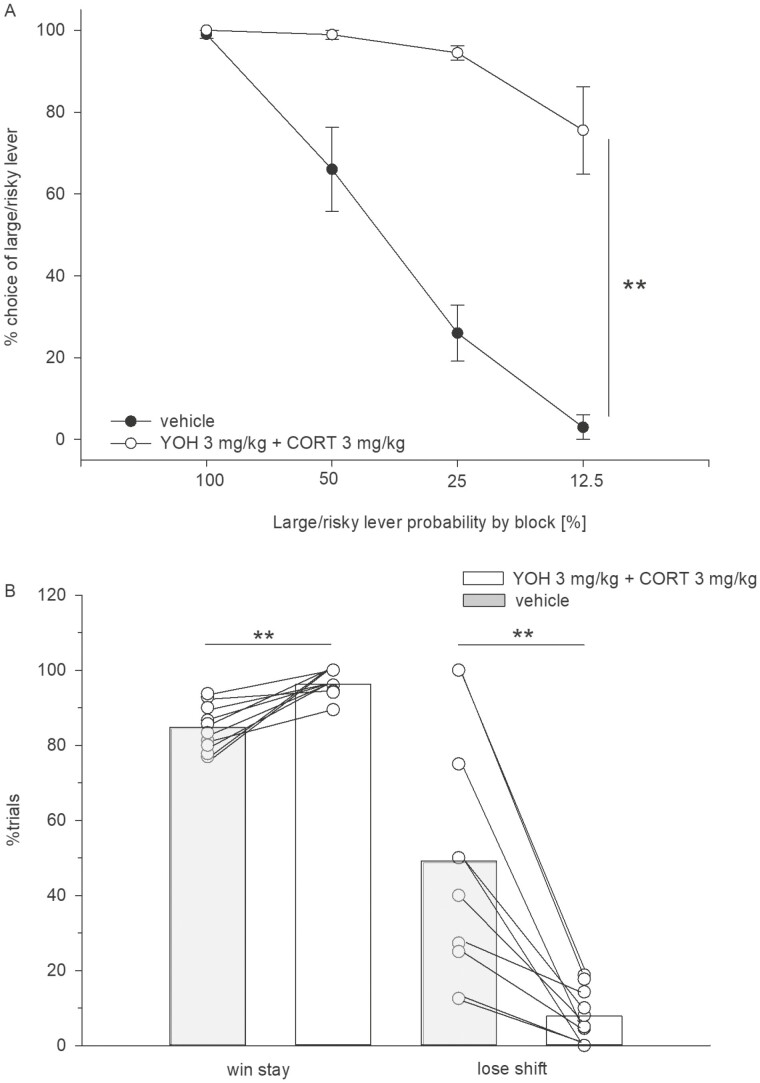
(A) Effects of co-administered yohimbine (3 mg/kg, i.p.) and corticosterone (3 mg/kg, i.p.) vs vehicle on probabilistic choice. Mean (±SEM) percentages of large/risky lever choices per session are shown across decreasing probabilities to obtain the large/risky reward within 1 session (n = 9). **P < .01 (significant main effect of treatment, ANOVA). (B) Mean proportion (±SEM) of large/risky lever choices after risky wins among all rewarded large/risky lever choices (“win-stay”) and mean proportion (±SEM) of small/certain lever choices after risky losses among all small/certain lever choices (“lose-shift”). **P < .01, *t* test.

Then, we tested for combined effects of yohimbine and corticosterone. Because both doses of corticosterone had no effects by its own and risk-taking in rodents positively correlates with plasma corticosterone levels (Rodgers et al., 1999), we only tested the higher (3 mg/kg) not lower dose (1 mg/kg) of corticosterone in combination with yohimbine. Co-administration of both drugs at 3 mg/kg (each) increased the large/risky lever preference (Figure 3A). Data from 1 animal that failed to lever press was not included. An ANOVA showed a main effect of treatment (F_(1,8)_ = 119.6, *P* < .001), a significant effect of trial block (F_(3,24)_ = 77.16, *P* < .001), and a treatment × block interaction (F_(3,24)_ = 17.11, *P* < .001). Moreover, a statistical analysis on win-stay ratio data revealed a significant treatment effect (*t* test *t*_8_ = 4.64; *P* < .01) (Figure 3B). Also, lose-shift ratios differed as a function of treatment (*t* test, *t*_8_ = 3.96; *P* < .01).

An additional analysis on large/risky lever preferences in different treatment groups that received single administration of yohimbine (3 mg/kg) or co-administration of yohimbine and corticosterone (3 mg/kg, each) indicated no significant difference. A mixed ANOVA on risky/large reward lever presses with treatment/groups yohimbine + corticosterone vs yohimbine alone as between-subjects factor and block as repeated-measures factor revealed no main effect of treatment/group (F_(1,18)_ = 1.58, n.s.), a significant effect of trial block (F_(3,54)_ = 12.23, *P* < .001), and no treatment/group × block interaction (F_(3,54)_ = 0.24, n.s.).

### Experiment 2: Effects of Intra-PL D1 Receptor Blockade on Risky Choice

**Figure 4.**
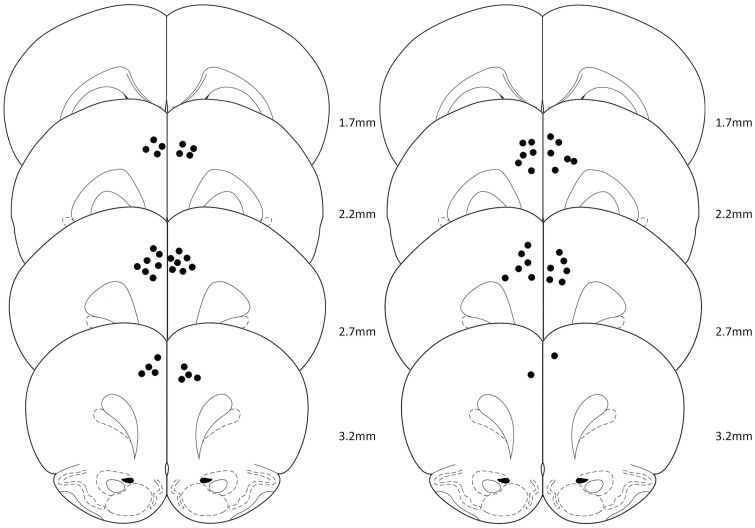
Cannula placements. The schematics depict the location of the injection cannula tips [(A) subgroup I, n = 15; (B) subgroup II; n = 13]. Data of 2 animals in subgroup II (not shown in B) were excluded due to cannula misplacement. Plates are modified from the atlas of Paxinos and Watson (1997). Numbers beside each plate correspond to millimeters anterior to bregma.

**Figure 5.**
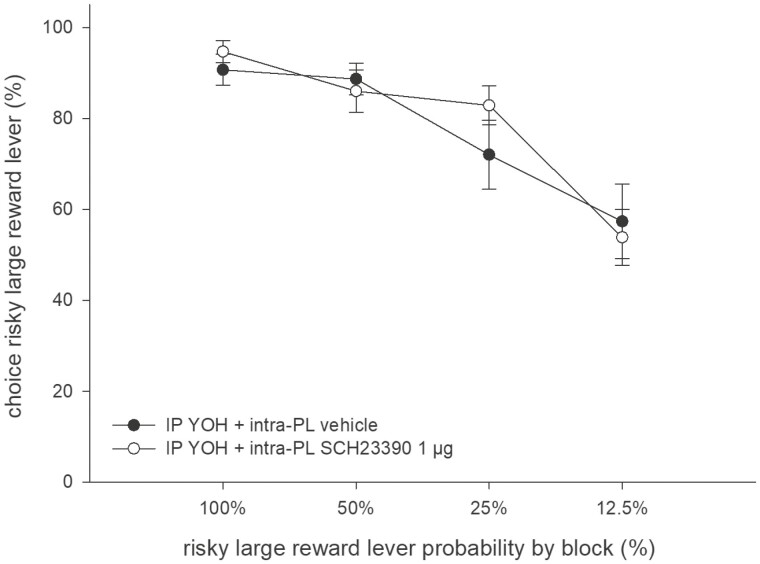
Effects of intra-PL SCH23390 (1.0 µg) vs vehicle on probabilistic choice in animals pretreated with yohimbine (3 mg/kg, i.p.). Mean (±SEM) percentages of large/risky lever choices per session are shown across decreasing probabilities to obtain the large/risky reward within 1 session (n = 15).

In animals pretreated with systemic yohimbine (3 mg/kg), intra-PL microinfusion of SCH23390 (1.0 µg) did not alter the preference for the large/risky lever compared with intra-PL microinfusion of vehicle (Figures 4A and 5). An ANOVA revealed no main effect of treatment (F_(1,12)_ = 0.25, n.s.), a main effect of trial block (F_(3,69)_ = 26.14, *P* < .01), and no treatment × block interaction (F_(3,36)_ = 0.78, n.s.).

**Figure 6.**
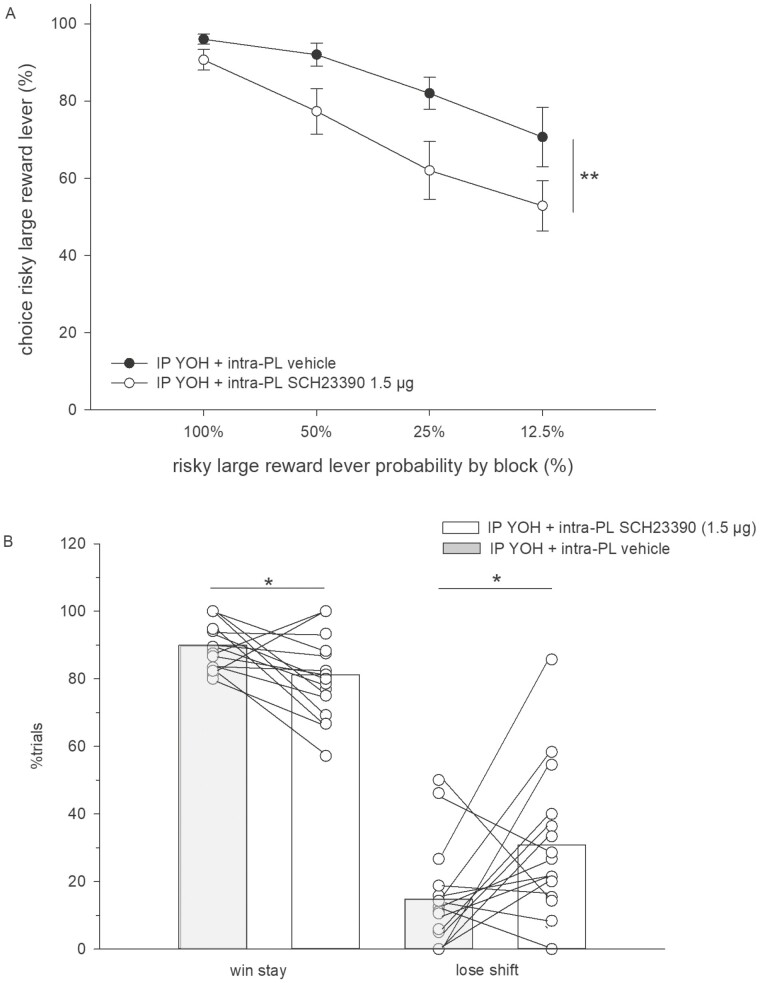
(A) Effects of intra-PL SCH23390 (1.5 µg) vs vehicle on probabilistic choice in animals pretreated with yohimbine (3 mg/kg, i.p.). Mean (±SEM) percentages of large/risky lever choices per session are shown across decreasing probabilities to obtain the large/risky reward within 1 session (n = 15). **P < .01 (significant main effect of treatment, ANOVA). (B) Mean proportion (±SEM) of large/risky lever choices after risky wins among all rewarded large/risky lever choices (“win-stay”) and mean proportion (±SEM) of small/certain lever choices after risky losses among all small/certain lever choices (“lose-shift”). *P < .05, *t* test.

By contrast, in yohimbine-pretreated animals (3 mg/kg), intra-PL microinfusion of SCH23390 at a higher dose (1.5 µg) reduced the preference for the large/risky lever compared with intra-PL microinfusion of vehicle (Figures 4A and 6A). An ANOVA revealed a main effect of treatment (F_(1,13)_ = 11.63, *P < *.01) and trial block (F_(3,39)_ = 22.61, *P* < .01) and no treatment × block interaction (F_(3,39)_ = 1.36, n.s.). In addition, in rats with intra-PL microinfusion of SCH23390, the number of win-stay trials was reduced (*t*_*12*_ = 2.46, *P* < .05), and the number of lose-shift trials (*t*_*12*_ = 2.31, *P* < .05) was increased compared with rats with intra-PL vehicle infusion (Figure 6B).

**Figure 7.**
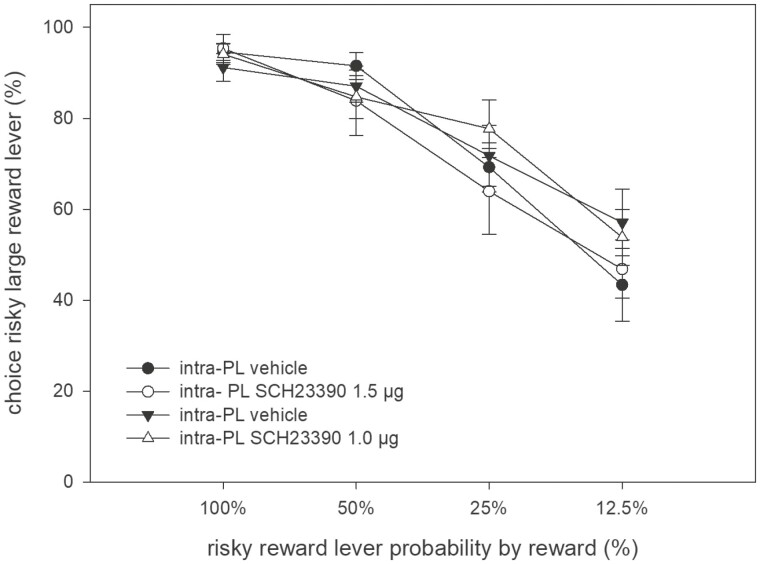
Effects of intra-PL infusions of SCH23390 (1.0; 1.5 µg) vs respective vehicle infusions (tested in 2 sessions) on probabilistic choice. Mean (±SEM) percentages of large/risky lever choices per session are shown across decreasing probabilities to obtain the large/risky reward within 1 session (n = 13).

In animals not pretreated with yohimbine, intra-PL microinfusions of SCH23390 (1.0, 1.5 µg) did not alter the preference for the large/risky lever compared with respective intra-PL microinfusions of vehicle (Figure 4B, 7). An ANOVA shows no main effect of treatment (F_(3,33)_ = 0.85, n.s.), a main effect of trial block (F_(3,33)_ = 47, 31, *P* < .01), and no treatment × block interaction (F_(9,99)_ = 1.27, n.s.).

## DISCUSSION

Using a risk discounting task, we showed that systemic yohimbine increased the preference for large/risky reward. By contrast, single systemic administration of corticosterone did not significantly promote risky choice and co-administration of corticosterone did not enhance the effects of yohimbine on risky choice. Moreover, our data reveal that the increased preference for large/risky reward induced by systemic yohimbine was lowered by concurrent intra-PL D1 receptor blockade. This latter finding suggests that enhanced risky choice elicited by the acute pharmacological stressor yohimbine involves D1 receptor activation in the PL.

### Pharmacological Stressors and Risk Discounting

Experiment 1 reveals that, relative to vehicle controls, systemic yohimbine at a behaviorally active dose (Packard and Wingard, 2004; Nair et al., 2011; Chen et al., 2015) markedly enhanced the preference for the large/risky lever and reduced the number of certain choices after risky loss. Because rats were group-housed and received 15 g of laboratory chow per animal and day given per cage, we cannot completely exclude the possibility that small differences in individual body weight gain could influence instrumental performance. However, such an effect, if any, might be small, because in previous studies we did not notice correlations between instrumental performance and body weight.

Both effects of yohimbine seen here correspond well with those of a previous study using an almost identical risk discounting task (Montes et al., 2015). However, it is important to note that in this task, acute restraint stress did not alter risky choice, whereas intracerebral administration of corticotropin releasing factor did (Bryce et al., 2020). Our data also demonstrate that systemic corticosterone at behaviorally active doses (Roozendaal et al., 2004; Sadowski et al., 2009) did not alter risk discounting and, after co-administration, did not significantly enhance the effects of yohimbine on risk discounting. In contrast, in other risk tasks, systemic injections of corticosterone increased risky choice in rats (Koot et al., 2013). Moreover, acute cortisol levels were positively correlated with risk-taking in humans (Cueva et al., 2015; Kluen et al., 2017). Collectively, these variable findings suggest that acute stressor effects on risky choice are markedly influenced by several parameters, such as stressor identity (e.g., naturalistic/nonpharmacological vs pharmacological stressors) and task structure (see Braun and Hauber, 2013; Bryce et al., 2020). For instance, rat and human studies employ multiple risk paradigms that involve in part disparate cognitive and neurochemical mechanisms that may differ in their sensitivity to pharmacological stressors such as yohimbine or corticosterone or naturalistic stressors such as predator exposure or restraint. Future studies should attempt to disentangle the underlying mechanisms in more detail. For example, in view of studies demonstrating increased risky choice in a gambling task in rats exposed to inescapable foot shock (Nobrega et al., 2016), it seems critical to test whether this acute stressor is also able to enhance risky choice in the task used here and, in addition, whether such effects could be modified by concurrent administration of NA drugs. Such experiments could provide further insight as to which types of naturalistic/nonpharmacological stressors are able to influence risky choice as tested in our study and whether such effects involve enhanced NA activity. Taken together so far, our data demonstrate that yohimbine, but not corticosterone, can increase risky choice. This former finding supports the notion that enhanced NA activity as induced by yohimbine (Aghajanian and VanderMaelen, 1982) provides a key mechanism through which acute stressors alter risk-based decision-making (Montes et al., 2015). Moreover, there is considerable preclinical evidence that acute stress exposure is associated with an increased firing of the locus coeruleus as well as an increased NA release in brain regions receiving NA innervation (Bremner et al., 1996a). Also, clinical studies revealed that acute stressors, such as public speaking, are associated with increased NA levels (Bremner et al., 1996b).

### PL D1 Receptor Activity and Risk Discounting

As noted above, the neurochemical mechanisms and brain areas through which yohimbine impairs cognitive function are not well understood. Given the prominent role of PL D1 receptors in cognition and acute stress (Robbins, 2005; Arnsten, 2015), we asked in Experiment 2 whether the effects of yohimbine on risky choice seen in Experiment 1 could be mediated by PL D1 receptors. The results first demonstrate that intra-PL SCH23390 (1.0; 1.5 µg) did not significantly alter risk discounting in animals without yohimbine pretreatment. This finding suggests that in nonstressed animals D1 receptor activity has a limited influence on risky choice as tested here. At variance with this notion, a previous study revealed that medial prefrontal microinfusion of SCH23390 reduced the large/risky reward lever preference (St Onge et al., 2011). These discrepant findings may be predominantly accounted for by differential localization of prefrontal subregions targeted by microinfusions, which may vary in terms of their behavioral function. Despite some overlap, our microinfusions are more dorso-caudal and involve the pregenual anterior cingulate cortex and dorsal prelimbic areas, which have been found to support in part other behavioral functions than the ventral prelimbic and infralimbic cortex targeted in the study by St Onge et al. (2011) (see e.g., Diehl and Reddish, 2023). For instance, the anterior cingulate cortex and dorsal prelimbic areas are strongly active during acute decision-making in highly complex tasks (Diehl and Reddish, 2023) and play a role in adapting action plans and shifting to new strategies (Brockett et al., 2020). By contrast, ventral prefrontal areas are more associated with goal-directed decision-making in tasks in which performance relies on well learned action-outcome associations and knowledge of the task structure (Killcross and Coutureau, 2003). Moreover, the dorsal and ventral prelimbic areas differ in terms of their connectivity. For instance, the dorsal prelimbic areas have stronger cortical projections, whereas more ventral prelimbic areas have stronger projections to basal ganglia structures (Heidbreder and Groenwegen, 2003). Given that D1 receptors modulate these behavioral functions, it is conceivable that microinfusions of SCH23390 targeted to more ventral prefrontal subregions (St Onge et al. 2011) interfered with retrieval of learned associations and resulted in altered task performance, whereas our infusions into the dorsal prelimbic area, which modulates of strategy shifts, did not interfere with task performance. Another methodological disparity between the study by St Onge et al. (2011) and our study relates to subtle differences in risk preferences between vehicle controls across studies that may explain in part the observed differences of effect sizes of SCH23390.

Importantly, the results of Experiment 2 further reveal that enhanced risky choice under yohimbine was significantly lowered by concurrent intra-PL infusion of the higher (1.5 µg), but not lower (1.0 µg), dose of SCH23390. Also, intra-PL infusion of SCH23390 (1.5 µg) counteracted yohimbine-induced effects on lose-shift/win-stay behavior. These findings suggest that increased PL D1 receptor activity represents a major neurochemical mechanism underlying the effects of yohimbine on risky choice. Consistent with this view, activation of NA neurons in the locus coeruleus not only increased NA but also DA release in several brain areas, including the prefrontal cortex (Tanda et al., 1996; Devoto and Flore, 2006). Also, yohimbine at a dose similar to that used here selectively increased DA levels in the prefrontal cortex but not in the nucleus accumbens (Chen et al., 2015). Moreover, dorsal, but not ventral, prefrontal SCH23390 microinfusions decreased stress-induced reinstatement of food seeking induced by systemic yohimbine (Nair et al., 2011). However, at variance with our account, a previous study found that pharmacological stimulation of prefrontal D1 receptors did not enhance risky choice (St Onge et al., 2011). However, as noted above, the different microinfusion targets may account for this discrepancy. By contrast, stimulation of D1 receptors in more ventral prefrontal subregions promoted risk-taking in a rat task in which external cues inform about risk (Yang et al., 2022). Also, temporal overexpression of prefrontal D1 receptors enhanced risky choice in mice tested in a rodent Iowa gambling task (Beyer et al., 2021). Collectively, these findings further confirm the notion that the involvement of prefrontal D1 receptors in risky choice may be critically influenced by task structure and the targeted prefrontal subregion. Moreover, it is conceivable that the effects of yohimbine seen here may involve PL D1 receptor activation in concert with other neurochemical processes within the PL and in other brain areas. For instance, in prefrontal neurons, DA and NA signaling pathways acted together to potentiate each other’s postsynaptic actions (Arnsten, 2009; Xing et al., 2016), and a simultaneous blockade of DA and NA reuptake promoted disadvantageous risky choice (Baarendse et al., 2013). Also, it is possible that yohimbine effects in other brain regions known to modulate risky choice, such as the amygdala, could synergize with enhanced PL D1 receptor activity to increase risky choice.

### Acute Stress and Risky Choice

There is ample evidence that acute stress in humans alters decision-making under risk. A meta-analysis involving 1829 participants suggests that exposure to acute stressors leads to more risk-taking relative to nonstressor conditions (Starcke and Brand, 2016). Moreover, clinical studies provide correlative evidence for a role of catecholamines in modulating risk-based decision-making. For instance, in healthy humans, elevated catecholamine activity induced by methylphenidate increased risky choice (Campbell-Meiklejohn et al., 2012). Similarly, neuropharmacological data from laboratory animals highlight that acute stress impairs cognitive function by excessive activation of prefrontal D1 and α1 receptors (Arnsten, 2009). Our rodent data provide causal evidence that stimulation of PL D1 receptors may represent a neurochemical mechanism by which the acute pharmacological stressor yohimbine, and possibly nonpharmacological stressors as well, promote risky choice.

We are aware that our study has several limitations. First, although yohimbine is a widely used pharmacological tool due to its well-documented stress-inducing properties in humans and laboratory animals, it may only partially mimic the complex behavioral and physiological effects induced by acute naturalistic stressors (Schwabe et al., 2022). Second, given the discrepancies in task structures and prefrontal subregions targeted in the available rodent studies, the precise role of prefrontal subregions in risky choice needs to be addressed in more detail in systematic future studies, perhaps also using interventions with higher spatiotemporal specificity such as optogenetics. Third, we used only male rats and future studies should include both sexes to address sex differences in risky choice as observed in a number of previous studies in rats. For instance, in a risk-based decision-making task in which the risk of larger reward was associated with footshocks, males chose large, risky rewards more than females did (Orsini et al., 2016, 2021; Truckenbrod et al., 2023). Likewise, in a similar task as used here, males showed a higher preference than females for the large risky reward, specifically when the probability of obtaining a larger food reward was only 25% or 12.5% (Islas-Preciado et al., 2020). Together, these findings point to the possibility that in tasks as used in our study females are less risk prone than males and, in turn, more susceptible to risk-enhancing effects of pharmacological stressors such as yohimbine.

## Data Availability

The data underlying this article will be shared on reasonable request to the corresponding author.

## Notes

This work was supported by a grant from the Deutsche Forschungsgemeinschaft (DFG) to W.H. (HA2340/9-1).

## Interest Statement

None declared.
